# Evaluation of a Wellness Program For Affordable Senior Housing Residents

**DOI:** 10.1093/geroni/igaf122.3002

**Published:** 2025-12-31

**Authors:** Verena Cimarolli, Alisha Sanders, Molly Wylie, Robyn Stone

**Affiliations:** LeadingAge, Washington, District of Columbia, United States; Volunteers of America National Services, Washington, District of Columbia, United States; LeadingAge, Washington, District of Columbia, United States; LeadingAge, Washington, District of Columbia, United States

## Abstract

Volunteers of America National Services’ (VOANS) Aging with Options (AWO) program - funded by and developed in partnership with Parker Health Group - is designed to support wellness and improve chronic disease management for residents in affordable senior housing. The intervention, piloted at one housing site, provided in addition to an existing service coordinator, a wellness nurse and a community health worker to help engage residents in healthy behaviors and connect them with resources. The evaluation effort assessed participants’ perceptions of the program’s impact by implementing a resident survey (N = 69); and determined the program’s effect on health outcomes by analyzing service- and health-related data collected regularly by program staff (N = 99). Over 94% of AWO participants reported that the supports received from AWO program staff were helpful. The most frequently mentioned program benefits were an improved sense of health, enhanced well-being and safety, and greater social connection. Residents who developed a wellness plan - for which they identified health and wellness goals and steps to achieve these goals - reported experiencing positive changes such as eating healthier food, greater success controlling health conditions, and exercising more. Regression analyses conducted to identify service-related predictors of health outcomes six months after the program’s initiation showed that higher life optimism was associated with receiving more service instances of ‘follow-up with service providers.’ Further, better self-rated physical health was associated with receiving more service instances of ‘exercise/physical fitness’, ‘socialization’, and ‘encouragement to attend event/program.’” Evaluation findings underscore the program’s benefits for resident health and well-being.

